# Beclin1 circulating level as predictor of carotid intima-media thickness in patients with type 2 diabetes mellitus

**DOI:** 10.1097/MD.0000000000026630

**Published:** 2021-07-16

**Authors:** Mervat Naguib, Aya Tarabay, Nashwa ElSaraf, Lila Rashed, Amr ElMeligy

**Affiliations:** a Diabetes and Endocrinology Unite, Faculty of Medicine Kasr Al-Ainy Hospital, Cairo University, Egypt; b Internal Medicine Department, Faculty of Medicine Kasr Al-Ainy Hospital, Cairo University, Egypt; c Department of Biochemistry, Faculty of Medicine, Cairo University, Egypt.

**Keywords:** autophagy, Beclin-1, carotid intima-media thickness, diabetes

## Abstract

Type 2 diabetes (T2DM) represents a major risk factor for atherosclerosis that is the underlying cause of most cardiovascular diseases. Identifying reliable predictive biomarkers are needed to improve the long-term outcome in diabetic patients. Autophagy plays a pivotal role in the pathogenesis of atherosclerosis. Beclin1 is a key regulatory protein of autophagy and has been localized in human atherosclerotic lesions. However, the relation of serum level of Beclin1 and atherosclerosis in patients with diabetes has not been clarified yet.

To assess the relationship between serum level of Beclin1 and carotid intima-media thickness (CIMT) in patients with T2DM.

In this case-control study participants were recruited from tertiary care hospitals in Egypt. The study enrolled 50 patients with T2DM and 25 healthy subjects between January, 2019 and January, 2020. Age, gender, and body mass index were recorded for all subjects. Laboratory works up including glycated hemoglobin, lipid panel, and serum Beclin1 (by enzyme-linked immunosorbent assay) were measured. CIMT was assessed by color Doppler. Comparisons between patients and the control group were done using analysis of variance and Chi-square test. Correlations between CIMT and Beclin1 level and different variables were done using the Pearson correlation coefficient. Receiver operator characteristic curve was constructed with the area under curve analysis performed to detect the best cutoff value of Beclin1 for detection of CIMT > 0.05 cm.

The level of Beclin1 in the patient group was significantly lower compared with that in the control group (1.28 ± 0.51 vs 5.24 ± 1.22 ng/dL, *P* < .001). The level of Beclin1 apparently decreased in the higher CIMT group in T2DM patients. Serum Beclin1 levels were negatively correlated with CIMT (*r* = –0.762; *P* < .001), low-density lipoprotein-cholesterol (*r* = −0.283; *P* = .04), and triglycerides (*r* = −0.350; *P* = .01) but positively correlated with high-density lipoprotein-cholesterol (*r* = 0.491; *P* < .001) in patients with T2DM. Beclin1 level >2.2 ng/dL was an accurate predictor of CIMT >0.05 cm with an area under the curve value of 0.997, 93.9% sensitivity, and 100% specificity.

Beclin1 levels were negatively correlated with atherosclerotic load in patients with T2DM and it may be considered as a promising diagnostic and therapeutic target.

## 1 Introduction

Diabetes is a major health problem worldwide and is a well-known risk factor for multiple micro and macrovascular complications. Macroangiopathy secondary to diabetes affects up to 32.2% of the patients with diabetes and typically occurs earlier, with more severe, and wider distribution than in individuals without diabetes.^[1,2]^ Atherosclerosis may cause cerebral vascular disease, ischemic heart disease, and peripheral arterial disease, which are major causes of morbidity and mortality in patients with diabetes.^[3]^

The main pathological mechanisms in diabetes-associated macroangiopathy involve a disturbance in the endothelial function and alternations in vascular smooth muscles. Although the presence of several metabolic abnormalities implicated in the progression of atherosclerosis in patients with diabetes, there are different mechanisms and pathological features that are distinct at the onset of atherosclerotic changes via engaging different cells from the vascular wall and circulatory cells.^[3]^ Efforts are recently directed to the detection of cellular compensatory mechanisms against atherosclerotic changes to restore cell function or hold severe vasculopathy.^[4]^

Autophagy is an intracellular self-cleaning process that disposes damaged organelles and injured cytoplasmic components through double-lipid bilayer membrane vesicles out the target cells. It is a vital process that is important to maintain cellular homeostasis that is activated following cellular stresses such as nutrient limiting conditions or various pathogens and can protect the cell from apoptosis.^[5]^ Recent researches demonstrate the important and protective role of autophagy in the endothelial cells and smooth muscle cells’ defense against different injuries.^[6]^

Autophagic process was found to be involved in the pathogenesis of type 2 diabetes mellitus (T2DM) disease, and many diabetes-related complications.^[7]^ Autophagy was found to regulate the normal function of pancreatic beta cells moreover; stimulated autophagy acts as an important defensive mechanism against oxidative stress on insulin target sites such as the liver, adipose tissue, and skeletal muscle.^[8,9]^

Beclin1, a highly conserved eukaryotic protein, has a major regulatory role in autophagy. It is a component of the phosphatidylinositol-3-kinase complex which mediates vesicle trafficking thereby inducing autophagy.^[10]^ Beclin1 dysfunction has been incriminated in many disorders, including malignancy, diabetes, and neurodegenerative diseases.^[11,12]^

Although defective autophagy could be one of the causes for instability of carotid atheroma plaques, factors affecting autophagy can act differentially in different cell types and different stages of the developed plaque.^[13]^ Researches investigating the relation between Beclin1 and atherosclerosis are few and contradictory; moreover, they were experimental and investigated Beclin1 at the cellular level.^[14,15]^ Beclin1 has been recently immunolocalized in human carotid and main cerebral artery plaques.^[16]^ Recently, it has been documented that autophagy inhibition through the suppression of Beclin-1 stimulates the secretion of IL-1β.^[17]^ However, when a Beclin-1 peptide was administered to pro-atherogenic ApoE-null mice, Beclin-1 peptide did not inhibit the development of atherosclerosis.^[18]^

To the best of our knowledge, no previous human studies assessed the serum level of Beclin1 in relation to atherosclerosis. We assumed that serum Beclin1 level, as a marker of autophagy may be decreased in diabetic patients with atherosclerosis. So, we evaluated serum Beclin1 level in a group of patients with T2DM and explored its relation to carotid intima-media thickness (CIMT) as a marker of atherosclerosis in this group of patients.

## 2 Material and methods

### 2.1 Study design

This study was a case-control study conducted between January, 2019 and January, 2020. Adults who underwent voluntary health evaluation at the diabetic center, Kasr Al Ainy Hospital, Cairo, Egypt with T2DM and their age between 40 and 60 years were included in the analysis. We assigned 50 patients with T2DM and 25 age and sex healthy volunteers from the employees at the same hospital as control.

Patients with a diagnosis of T2DM based on American Diabetes Association criteria were eligible for inclusion in this study. Exclusion criteria included patients with 1) diabetes other than T2DM; 2) diabetic kidney disease as diagnosed by estimated glomerular filtration rate and urinary albumin creatinine ratio; 3) history of smoking; and 4) history of ischemic heart disease or cerebrovascular disease. Clinical data obtained included age, sex, waist circumference, body mass index [weight (kg)/height (m^2^)], and duration of diabetes. Blood samples were collected from each individual after a period of 10 to 12 hours fasting for measurement of fasting blood glucose, glycated hemoglobin (HbA1C), total cholesterol (TC), high-density lipoprotein (HDL), low-density lipoprotein (LDL), triglycerides (TG), and serum Beclin1.

All subjects provided informed consent to participate in this study. The study protocol and procedures conform to the ethical guidelines of the 1975 declaration of Helsinki. Review board of Kasr Al Ainy hospital approved the study.

### 2.2 Sample size calculation

Using the following equation, the calculated sample size is 75 patients with T2DM. According to Cohn criteria, this number will be large enough to detect a medium correlation (*r* = 0.32) between Beclin-1 and CIMT in patients with T2DM, at a 95% level of confidence and an 80% power of the study.

Total sample size = N = [(*Z*α + *Z*β)/*C*]2 + 3^[19]^

where:

The standard normal deviate for α = *Z*α = 1.96

The standard normal deviate for β = *Z*β = 0.84

Correlation Coefficient for medium correlation = 0.32^[20]^

*C* = 0.5 × ln[(1 + *r*)/(1 − *r*)] = 0.332

### 2.3 Biochemical assay

Eight milliliter of blood were collected from each candidate and divided as follows: 2 mL were extracted by aseptic venipuncture to ethylenediaminetetraacetic acid (EDTA) containing vacutainer tubes for the Beclin-1 assay; 2 mL of blood were extracted to EDTA tube for assaying HbA1C; 2 mL of blood were reserved in a red-topped serum separator tube, serum was collected by centrifugation, samples were examined for lipid profile, and 2 mL of blood was extracted into fluoride tube for determining serum fasting glucose. Fasting blood glucose was measured using the enzymatic spectrophotometric method. The HbA1c measurement was based on a turbid-metric inhibition immunoassay principle. TC concentration was measured using a polychromatic endpoint technique. TG and HDL levels were measured using a bichromatic endpoint technique. LDL-cholesterol was calculated according to the Friedewald equation from measured values of total cholesterol, TG, and HDL-cholesterol according to the relationship: LDL = TC − HDL − TG/5.^[21]^ Clinical chemistry analysis was done on Dimension RxL Max (Siemens, USA).

### 2.4 Beclin-1 assay principle

Serum Beclin1 level was measured using enzyme-linked immunosorbent assay kit based on Beclin1 antibody-Beclin1 antigen interactions (immunosorbency) and an horseradish peroxidase colorimetric detection system to detect Beclin 1 antigen targets in samples (MyBioSource, Inc, CA, USA) according to the manufacturer's instructions. Samples were collected in EDTA tubes; centrifugation was performed for 15 minutes at 1000×*g* at 2 to 8°C within 30 minutes of collection and samples stored at −80°C. Absorbance measurements were carried out in a microplate reader at 450 nm and the concentrations were calculated using a standard curve.

### 2.5 CIMT measurement

CIMT was measured in our study by B-mode duplex ultrasound modality. All participants were examined by a high-frequency 7.5 MHz linear probe of an ATl-HDL 5000 machine. Linear scanning of the right and left carotid arteries were done. CIMT was identified as the distance between intima-lumen line and media-adventitia borders.^[22]^ Three measurements were taken from the anterior and posterior walls of the common carotid artery, the carotid bifurcation, and the extracranial portion of the internal carotid artery. The average of both sides’ measurements was calculated. A carotid plaque was described as a focal thickening ≥1.5 mm.^[22]^ Doppler was done by 1 experienced physician blinded to their clinical condition.

### 2.6 Statistical analysis

Data were coded and entered using the statistical package (SPSS) version 25 (IBM Corp., Armonk, NY, USA). Measurement data are presented as mean and standard deviation for quantitative variables and frequencies and percentages for categorical variables. Comparisons were done using analysis of variance and Chi-square test. Correlations between quantitative variables were done using the Pearson correlation coefficient. Data tested for normality and proved to be normally distributed. Then, stepwise regression for selection of independent predictors of CIMT and Beclin1 in cases was performed and R-squared was measured to test how close the data are to the fitted regression line. Receiver operator characteristic curve was constructed to determine the sensitivity and specificity to the sensitivity and specificity of Beclin-1 for detection of CIMT >0.05 cm. *P* values <0.05 were considered statistically significant.

## 3 Results

### 3.1 Baseline characteristics of different groups

A total of 75 patients with T2DM (50 patients with T2DM and 25 healthy age and sex-matched controls) were enrolled in this study. Patients with T2DM were obese with mean body mass index (31.50 ± 6.01) kg/m^2^and 60% of them were hypertensive. The mean HbA1c was elevated (9.0 ± 1.7; 74.9 ± 8.5) (%, mmoL/moL) and the cholesterol, TG, and CIMT were significantly higher in patients with diabetes compared to the control group. Table 1 shows clinical characteristics and laboratory parameters of T2DM patients and the control group.

**Table 1:** Clinical and laboratory parameters of patients with T2DM and healthy subjects.

Variable	Patients with T2DM n = 50	Healthy subjects n = 25	*P* value^∗^
Age (mean/yrs)	49.18 ± 6.64	46.28 ± 6.37	.07
Gender (male/female) (%)	25/25	12/13	.87
BMI (kg/m^2^)	31.50 ± 6.01	28.72 ± 2.51	**.006**
Waist circumference (cm)	108.87 ± 10.25	101.93 ± 8.06	**<.001**
Fasting glucose (mg/dL)	180.88 ± 65.52	85.21 ± 8.35	**<.001**
HbA1c (%, mmoL/moL)	9.0 ± 1.7 (74.9 ± 8.5)	4.6 ± 0.4 (26.8 ± 2.3)	**<.001**
Triglygerides (mg/dL)	195.66 ± 38.77	106.92 ± 18.88	**<.001**
Total Cholesterol (mg/dL	254.30 ± 45.48	150.12 ± 12.98	**<.001**
HDL (mg/dL)	36.42 ± 5.06	59.32 ± 5.19	**<.001**
LDL (mg/dL)	181.34 ± 51.95	68.44 ± 15.44	**<.001**
Serum Beclin1 (ng/dL)	1.28 ± 0.51	5.24 ± 1.22	**<.001**
CIMT (cm)	0.074 ± 0.012	0.040 ± 0.0004	**<.001**

Values are mean ± SD. BMI = body mass index, CIMT = carotid-intima media thickness, HbA1C = glycated hemoglobin, HDL = high-density lipoprotein, LDL = low-density lipoprotein.

∗ *P* < .05 is considered considered statistically significant.

### 3.2 Serum Beclin1 levels in patients with T2DM and its correlation with different parameters

**Figure 1:**
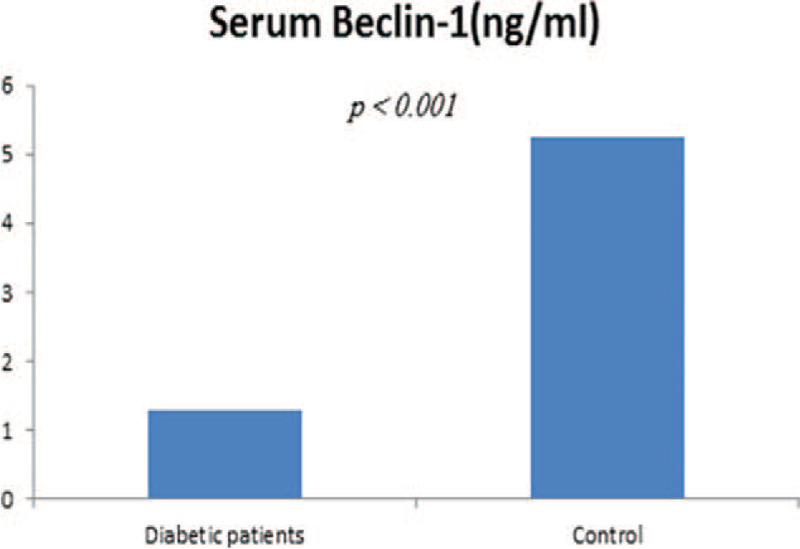
Serum level of Beclin1 was significantly lower in patients with T2DM compared to control subjects (1.28 ± 0.51 vs 5.24 ± 1.22 ng/dL, respectively, *P* < .001). T2DM = type 2 diabetes.

**Figure 2:**
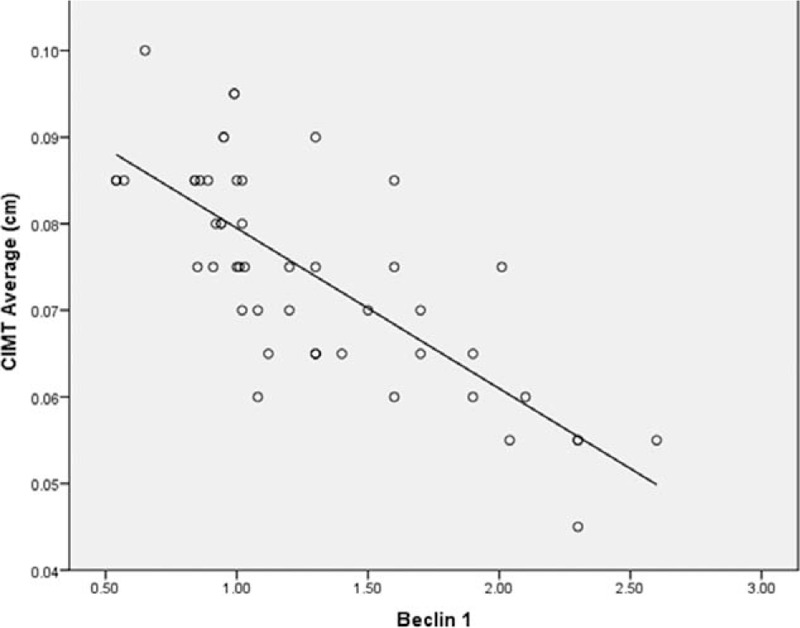
Correlation analysis of serum Beclin1 level and CIMT in patients with T2DM. There was a significant negative correlation between serum Beclin1 and CIMT (*r* = −0.762, *P* < .001). CIMT = carotid intima-media thickness, T2DM = type 2 diabetes.

Patients with T2DM had statistically significant lower serum Beclin1 compared to control subjects (1.28 ± 0.51 vs 5.24 ± 1.22 ng/dL, respectively; *P* < .001) (Fig. 1). Beclin1 protein level was negatively correlated to TG (*r* = –0.350, 95% CI: −0.573% to −0.079%; *P* = .01), LDL (*r* = −0.283, 95% CI: −0.521% to −0.006%; *P* = .04), and CIMT (*r* = −0.762, 95% CI: −0.858% to −0.613%; *P* = .001) (Fig. 2), but positively correlated to HDL (*r* = −0.491, 95% CI: 0.246% to 0.677%; *P* = .01) (Table 2). However, Beclin1 level was not statistically significant difference in diabetic patients with and without hypertension (*P* = .63).

**Table 2:** Correlations of serum level of Beclin1 with different variables in diabetic patients with T2DM.

Parameter	*r*	*P* value	95% CI
Age (yrs)	0.027	.85	−0.253 to 0.303
^1^BMI (kg/m^2^)	0.110	.44	−0.174 to 0.377
^2^HbA1C	−0.189	.18	−0.444 to 0.094
^3^TG (mg/dL)	−0.350	.01	−0.573 to 0.079
Cholesterol (mg/dL)	−0.348	.01	−0.571 to 0.077
^4^LDL (mg/dL)	−0.283	.04	−0.521 to 0.006
^5^HDL (mg/dL)	0.491	<.001	0.246 to 0.677
^6^CIMT (cm)	−0.762	<.001	−0.858 to 0.613

Values are mean ± SD. BMI = body mass index, CIMT = carotid-intima media thickness, HbA1C = glycated hemoglobin, HDL-C = high-density lipoprotein cholesterol, LDL-C = low-density lipoprotein cholesterol, TG = triglycerides l. ^∗^*P* value <.05 is considered significant.

### 3.3 CIMT in patients with T2DM and its relation to different variables

In patients with T2DM, CIMT was significantly higher than healthy control (0.074 ± 0.012 vs 0.040 ± 0.0004 cm, respectively; *P* < .001). Carotid plaque was identified in only 8 patients. CIMT was significantly higher in males (0.07 ± 0.01 cm) than in females (0.08 ± 0.01 cm) (*P* = .02). CIMT was positively correlated to TG (*r* = −0.339, 95% CI: 0.067% to 0.564%; *P* = .01), LDL (*r* = 0.520, 95% CI: 0.282% to 0.697%; *P* < .001), but negatively correlated to HDL (*r* = −0.584, 95% CI: −0.742%, −0.365%; *P* < .001) and Beclin1 level (*r* = −0.491, 95% CI: −0.858%, −0.613%; *P* < .001) (Table 3).

**Table 3:** Correlations of CIMT with different variables in diabetic patients with T2DM.

Parameter	*r*	*P* value	95% CI
Age (yrs)	0.142	.32	−0.142 to 0.404
BMI (kg/m^2^)	−0.085	.56	−0.355 to 0.199
HbA1C	−0.189	.18	−0.167 to 0.383
TG (mg/dL)	0.339	.01	0.067 to 0.564
Cholesterol (mg/dL)	0.570	<.001	0.347 to 0.732
LDL-C (mg/dL)	0.520	<.001	0.282 to 0.697
HDL-C (mg/dL)	−0.584	<.001	−0.742 to 0.365
Beclin1 (ng/dL)	−0.762	<.001	−0.858 to 0.613

Values are mean ± SD. BMI = body mass index, CIMT = carotid-intima media thickness, HbA1C = glycated hemoglobin, HDL-C = high-density lipoprotein cholesterol, LDL-C = low-density lipoprotein cholesterol, T2DM = Type 2 diabetes, TG = triglycerides l. ^∗^*P* value <.05 is considered significant.

### 3.4 Regression analysis of determinants of CIMT

Normality was tested for by Shapiro–Wilk and all dependent and independent variables proved to be not deviated from the normal distribution. Residues in the model were tested for: a) No outliers tested by Std (standardised) residual: the minimum and maximum values do not exceed ±3; b) The data points were independent tested by Durbin–Watson estimate; c) The distribution of these residuals was normal with mean near 0; d) Constant variance was tested by Plot ZRESID (regression standardized residual) into the *Y*-axis and ZPRED (regression standardized predicted value) into the *X*-axis. Analysis of variance showed the “usefulness” of the linear regression model with serum level of Beclin 1 had a significant impact on CIMT *F* (1,48) = 66.29, *P* < .001.

Multiple linear regression analysis indicated that Beclin1 (β = −0.601; 95% CI, −0.018 to −0.011; *P* < .001), total cholesterol (β = 0.221; 95% CI, 0.000 to 0.000; *P* = .009), hypertension (β = 0.174; 95% CI, 0.001 to 0.008; *P* = .01), and male sex (β = 0.171; 95% CI, 0.001 to 0.008; *P* = .02) were significant predictors of CIMT in patients with diabetes.

### 3.5 Diagnostic performance of Beclin 1 as predictor of CIMT in patients with diabetes

**Figure 3:**
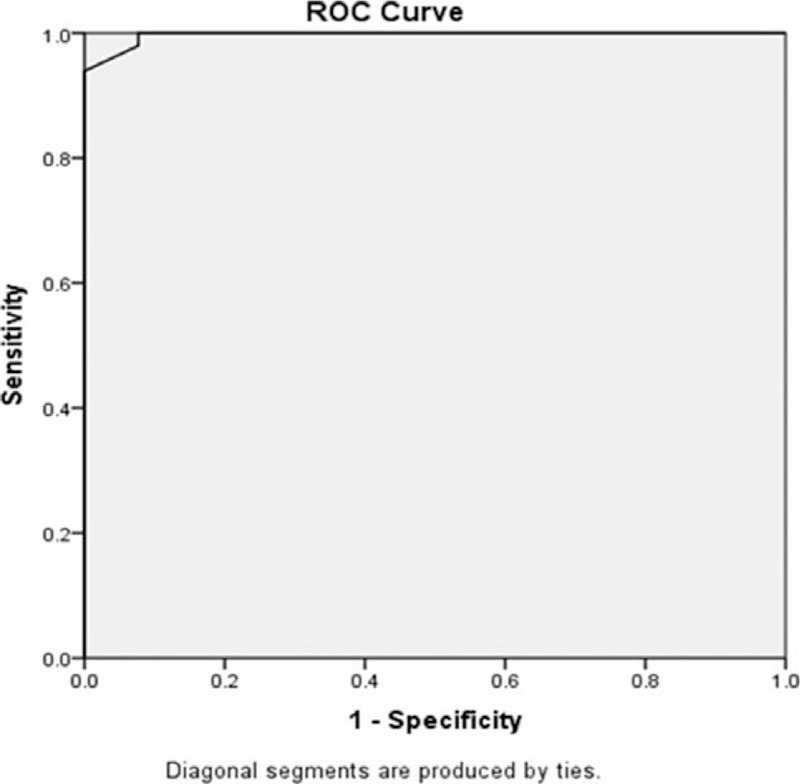
ROC curve indicating the predictive accuracy of Beclin1 for patients with CIMT >0.05 cm. (area under ROC, 0.997; 95% confidence interval, 0.990–1.000; *P* < .001). The best cutoff value was 2.2 ng/dL, with the best sensitivity (93.9%) and specificity (100%). CIMT = carotid intima-media thickness, ROC = receiver operator characteristic.

Receiver operator characteristic curve analysis for prediction of CIMT revealed that Beclin1 level >2.2 ng/dL was an accurate predictor of CIMT >0.05 cm with an area under the curve value of 0.997, the sensitivity of 93.9%, specificity of 100%, the positive predictive value of 100%, the negative predictive value of 89.66% and negative likelihood ratio of 0.06 (Fig. 3).

## 4 Discussion

The main result of our study was the negative association between the serum level of Beclin1, a key regulator of autophagy, and the CIMT in patients with T2DM. Furthermore, this association was independent of the traditional atherosclerotic risk factors. To the best of our knowledge, this is the first study that shows the associations between serum Beclin1 levels and carotid atherosclerosis in patients with diabetes.

Our data showed a significant reduction in Beclin1 level in patients with diabetes compared to healthy control. Hyperglycemia has been found to impair cellular autophagy.^[23]^ Furthermore; impaired autophagy has been reported to increase apoptosis of pancreatic beta cells after high-fat diet and high glucose diet in an experimental study.^[24]^ However, we found no significant correlation between the degrees of hyperglycemia as assessed by HbA1C level and Beclin1. These findings are approximately in line with a previous study of patients with diabetic kidney disease that showed no associations of Beclin 1 levels with HbA1c.^[25]^

In the current study, Beclin1 had significant positive correlations with atherogenic lipoproteins and negative associations with anti-atherogenic HDL cholesterol. Autophagy has been recently found to play role in the regulation of lipid metabolism including lipogenesis, lipolysis, fatty acid oxidation, ketogenesis, and cholesterol efflux that lead to the development of the atherogenic lipid profile with high TG and LDL and low HDL.^[26,27]^

Male sex, diabetes, hypertension, and dyslipidemia are well-known established risk factors of atherosclerosis.^[28,29]^ Our results were basically consistent with those of previous studies. Furthermore, the logistic regression analysis showed that serum Beclin1 was among the most important risk factors for CIMT in patients with T2DM.

The role of glycemic control in cardiovascular end points associated with T2DM is less clear, based on intervention studies.^[30]^ The effect of dyslipidemia might be more important in the process of atherosclerosis development in T2DM.^[31]^ This goes with the finding of our study as CIMT had a significant correlation with HDL and LDL but not with HbA1c.

A growing body of evidence implicates the role of autophagy dysfunction in the development of insulin resistance, lipid metabolism, inflammation, and endothelial dysfunction, which are the key players in the development of atherosclerosis.^[6,32,33]^ The absence of significant association between Beclin1 and glycated hemoglobin in the current study suggests that the association between autophagy and atherosclerosis is independent of hyperglycemia. Furthermore, the association between Beclin1 level and CIMT persisted when adjusting for other traditional risk factors indicating a direct effect of autophagy on the development of atherosclerosis.

Recent experimental studies reported that enhanced formation of Atg14L Beclin 1 Vps34 Vps15 complex, promotes autophagy-regulated lipid metabolism. Lipids are degraded to free cholesterol and fatty acids through autophagy.^[34]^ Another researcher found that inhibiting the activation of the mechanistic target of rapamycin activates autophagy and drives cholesterol efflux, thereby decreasing foam cell lipid accumulation,^[35]^ while another study suggested that lysosomal acid lipase mediated autophagy-mediated regulation of cholesterol efflux.^[36]^ Furthermore, mechanistic target of rapamycin inhibitor everolimus selectively depletes macrophages in atherosclerotic plaques by autophagy.^[37]^ These findings suggest that induction of autophagy is an essential strategy to reduce foam cell lipid accumulation and the prevention of atherosclerosis.

We also found that autophagy marker; Beclin1, can independently and accurately predict the CIMT in patients with diabetes. However, there are some limitations to the current study. First, because this study was case-control one, we cannot determine the causal relationships between lower Beclin1 levels and greater CIMT. Second, we did not assess the relation of Beclin1 level and the presence of plaque as we had a limited number of patients with carotid plaques in our study. Third, this study included a substantial number of patients on medications, requiring larger studies to separate the effects of such medications. Lastly, we did not measure microtubule-associated protein light chain 3 (LC3) which is the gold standard for studying autophagy also, we were not able to perform qRT-PCR and Western blot for Beclin-1 because of funding issues.

## 5 Conclusion

This study demonstrated an association between lower Beclin1 and greater CIMT in patients with T2DM, confirming the link between autophagy and atherosclerosis. Furthermore, this association was independent of other traditional risk factors of atherosclerosis.

## Acknowledgments

All authors acknowledge their gratitude to the staff members of diabetes unite for their help and support.

## Author contributions

**Conceptualization:** Mervat Naguib, Aya Tarabay, Nashwa ElSaraf, Lila Rashed, Amr ElMeligy.

**Data curation:** Aya Tarabay.

**Formal analysis:** Mervat Naguib, Aya Tarabay, Amr ElMeligy.

**Investigation:** Mervat Naguib, Aya Tarabay, Lila Rashed.

**Methodology:** Mervat Naguib, Nashwa ElSaraf.

**Supervision:** Mervat Naguib, Nashwa ElSaraf, Amr ElMeligy.

**Validation:** Mervat Naguib.

**Writing – original draft:** Mervat Naguib.

**Writing – review & editing:** Aya Tarabay, Nashwa ElSaraf, Lila Rashed, Amr ElMeligy.
